# A Study on the Efficacy of Empirical Antibiotic Therapy for Splenectomized Children with Fever

**DOI:** 10.25122/jml-2019-0086

**Published:** 2020

**Authors:** Gholamreza Bahoush, Marzieh Nojoomi

**Affiliations:** 1. Department of Pediatrics, Faculty of Medicine, Iran University of Medical Sciences, Tehran, Iran; Ali-Asghar Children Hospital,; 2. Ali-Asghar Children Hospital, Tehran, Iran; 3. Department of Community Medicine, Faculty of Medicine, Iran University of Medical Sciences, Tehran, Iran

**Keywords:** Spleen, splenectomy, antibiotic, acute infection, fever

## Abstract

Thalassemia represents a heterogeneous group of inherited diseases characterized by the lack or reduced production of hemoglobin β-chains. Many patients with thalassemia require splenectomy. What should be considered in the evaluation and management of candidates for splenectomy is to cover vaccination against infections such as pneumococci and the implementation of antibiotic prophylaxis. This study aimed to investigate the effect of the antibiotic type on the outcome of acute post-splenectomy infection in patients with thalassemia.

This investigation is a retrospective cohort study. One hundred fifty medical records of hemoglobinopathy patients who underwent splenectomy were collected from the Ali-Asghar Hospital, Tehran, Iran. SPSS v. 20 and SAS v. 1.9 were used to analyze the data.

A total of 150 patients that were vaccinated against post-splenectomy infections and were under antibiotic prophylaxis underwent splenectomy. The most commonly prescribed drugs were ceftriaxone or cefotaxime (132 cases, 88%), followed by ceftriaxone plus clindamycin (5.3%), ceftriaxone plus amikacin (3.3%), clindamycin (1.3%), vancomycin plus amikacin (0.7%), and others (1.3%). In terms of treatment outcomes, 143 cases (95.3%) were treated with the same antibiotics, and 4 (2.7%) experienced a changed antibiotic regimen with vancomycin.

The results show that perceptions of treatment for fever in splenectomized children need to be changed, and most of them do not require hospitalization and initiation of broad-spectrum antibiotics such as vancomycin for initially refractory cases, and can only be treated with daily intravenous ceftriaxone.

## Introduction

Thalassemia represents a heterogeneous group of inherited diseases characterized by the lack or reduced production of hemoglobin β-chains. The common pathophysiology bedrock involves increased destruction of red blood cells by the reticuloendothelial system, in particular by the spleen, resulting in its enlargement (splenomegaly) [1].

The spleen belongs to a network of cells and tissues, namely, the reticuloendothelial system found among all vertebrates. In adults, it is located in the left upper quadrant of the abdomen and is connected to the kidney and stomach by the splenorenal ligament and gastrosplenic ligament, respectively [2]. Spleen functions refer to the control of the quality of red blood cells by removing the old and impaired red blood cells in the red pulp, the production of antibodies in the white pulp, the removal of bacteria and blood cells that are covered by antibodies from the bloodstream, and ultimately, the storage of red cells and the increase of the above normal activities results in an increased size of the spleen [3].

Splenectomy is the only acceptable treatment for spleen injuries and, even in the presence of a variety of therapeutic modalities, such as laparoscopic splenectomy and partial splenectomy (PS), is considered as a standard therapeutic approach [4, 5]. Of course, splenic artery embolization is an option in some centers. Infections and thromboembolism are the effects of splenectomy [6]. However, the incidence of various types of infections, including sepsis, has been the most commonly reported postoperative event.

These bacteria cause an inflammatory infection called an overwhelming post-splenectomy infection (OPSI), which is associated with high mortality [7]. Infections result in splenectomy with two patterns, which include primary infections and delayed infections; the first one is mainly linked to surgery and its associated infections, and the second one is related to an impairment of the immune system due to the absence of the spleen. Post-splenectomy infections are the most common cause of morbidity during surgery and mainly include infections of the lower respiratory tract, intra-abdominal infections, wound infections, and non-specific infections requiring antibiotics [8]. Preventing infections in patients with splenectomy will require the following three strategies:

1. Patient education regarding the necessary care practices, symptoms of infection and infection prevention regimens;
2. An appropriate vaccination program;
3. Antibiotics should be used in a prophylactic fashion, covering pneumococcal infections. The increased risk of infection in these patients should be considered in the long-term [9]. Antibiotic prophylaxis using penicillin has already been shown to be highly effective in children with tuberculosis [10]. However, failure in antibiotic prophylaxis in the case of some antibiotics has also been well documented. The risk of sepsis after splenectomy is very high immediately after surgery; however, some cases of fulminant infections can also occur up to 20 years after surgery. The risk of sepsis in children under the age of 16 years and people over 50 will be higher [11]. Long-term penicillin therapy is useful, but sometimes accompanied by high antibiotic resistance and may even be associated with side effects such as allergies [12].

Some up-to-date scientific resources recommend immediate onset of broad-spectrum antibiotics that may include vancomycin. Therefore, this study aimed to investigate the effect of antibiotic prescriptions on the outcome of patients with acute post-splenectomy infection [13-15].

## Material and Methods

This investigation is a retrospective cohort study. The target population included all patients with hemoglobinopathy who had undergone spleen surgery and later had an infection post-splenectomy.

One hundred fifty medical records of hemoglobinopathy patients who underwent splenectomy were collected from the Asghar Hospital, Tehran, Iran, and relevant information including fever above 38°C, respiratory infections, age, gender, ceftriaxone or vancomycin use, and/or both, splenectomy time, and elapsed time from splenectomy were entered into the checklist and assessed analytically.

The definition of sepsis and the causative organism is shown in Table 1.

**Table 1:** The definition of sepsis and the causative organism.

**Infection**
A suspected or proven infection caused by any pathogen OR a clinical syndrome associated with a high probability of infection
SRIS (Systemic inflammatory response syndrome)↓
**Sepsis**
SRIS in the presence of infection
↓
**Sever Sepsis**
Sepsis plus one of the following:
(1) Cardiovascular dysfunction
(2) Acute respiratory distress syndrome
(3) Tow or more organ dysfunctions
↓
**Severe Shock**
Sepsis and cardiovascular organ dysfunctions

### Data analysis

The results for quantitative variables are presented as mean and standard deviation (mean ± SD). For the qualitative variables, the results were expressed as percentages. The comparison between quantitative variables was made using the t-test, and a Chi-square test was used to compare qualitative variables.

Data were analyzed using SPSS version 20 and SAS version 1.9. The significant level was considered to be less than 0.05.

### Ethical considerations

The participants were assured that information about their disease would remain confidential. They were also assured that their treatment would be routinely done if they would not accept the invitation to participate. Furthermore, the cost was not imposed on them in this study. All stages of the research were approved by the Ethics Committee of Iran University of Medical Sciences.

## Results

A total of 150 patients underwent splenectomy. In terms of sexual distribution, 45 subjects (30%) were men, and 105 (70%) were women. Regarding thalassemia type, 147 (98%) had thalassemia major, and 3 (2%) had thalassemia intermedia. The mean age of the patients at the time of diagnosis was 18.24 ± 6.51 years, ranging from 5 to 38 years. The mean age at splenectomy was 13.45 ± 5.82 years, ranging from 4-27 years. All patients were vaccinated against post-splenectomy infections, and therefore, vaccination coverage was reported as 100% in these patients.

**Figure 1:**
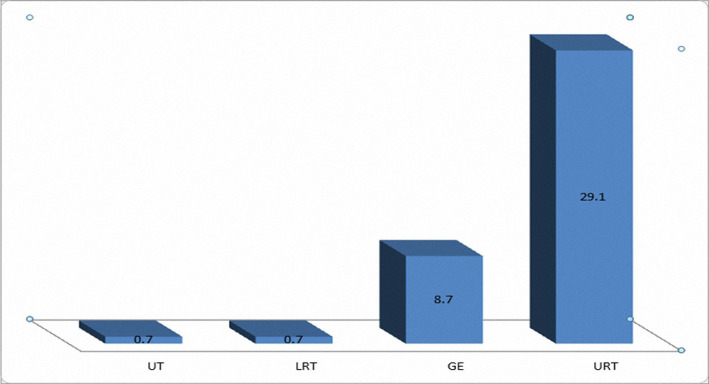
The location of the infection.

Overall, the most common infection was related to the upper respiratory tract (44 cases, 29.1%), followed by gastrointestinal infections (13 cases, 8.7%), lower respiratory tract infections (1 case, 0.7%), and urinary tract infections (1 case, 0.7%) (Figure 1). The other patients were suspected of sepsis as they had a fever without any localized symptom or sign.

In terms of the therapeutic approach, 34 cases (22.7%) were admitted, and the remaining 116 were outpatients (77.3%).

**Figure 2:**
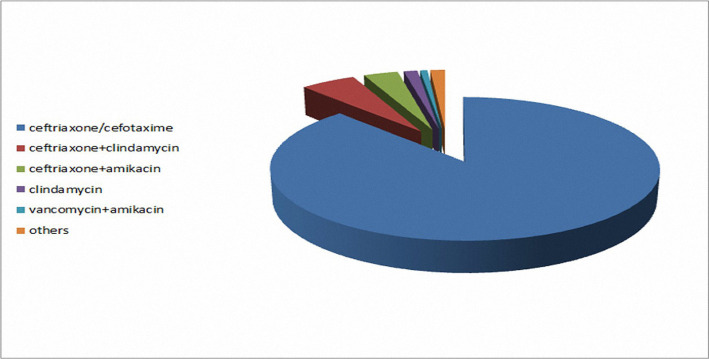
The most commonly administered antibiotics.

The prevalence of prescribed antibiotics is presented in Figure 2, and the results indicate that the most commonly prescribed drugs were ceftriaxone or cefotaxime (132 cases, 88%). Other instructions included ceftriaxone plus clindamycin in 8 cases (5.3%), ceftriaxone plus amikacin in 5 cases (3.3%), clindamycin in 2 cases (1.3%), vancomycin plus amikacin in 1 (0.7%) and others in 2 cases (1.3%).

Concerning treatment outcomes, 143 (95.3%) patients were treated with the first line of antibiotic therapy. Antibiotic changes (substituting with vancomycin) were done for 4 (2.7%), and antibiotic changes with antibiotics other than vancomycin were made for 3 (2%) patients.

In terms of the distribution of prescribed antibiotics in males and females, ceftriaxone was administered in 37 (82.2%) and 95 (90.5%), clindamycin alone in 0 (0%) and 2 cases (1.9%), ceftriaxone plus clindamycin was prescribed in 3 cases (6.7%) and 5 cases (4.8%), ceftriaxone plus amikacin in 4 cases (8.9%) and 1 (1%) vancomycin with amikacin in 0 (0%) and 1 case (1%), and the administration of other drugs occurred in 1 (2.2%) and 1 (1%) cases, respectively. There was no significant correlation between sex and prescription (P = 0.152).

Regarding the relationship between sex with the continuation of treatment lines in males and females, the continuation of the first line of treatment was found in 42 males (93.3%) and 101 females (96.2%), and the change of an antibiotic with vancomycin was seen in 2 males (4.4%) and 2 females (1.9%). Furthermore, the change of the antibiotic regimen with antibiotics other than vancomycin was considered in 1 (2.2%) and 2 (1.9%) males and females, but no relationship was found between gender and the therapeutic regimen (P = 0.669).

Our results showed no significant relationship between the type of drug regimen and the patient’s age (P = 0.942). Besides, there was no relationship between the age of patients and the change in the therapeutic regimen (P = 0.561).

The distribution of prescribed antibiotics according to the treatment method (hospitalization or outpatient) is shown in Table 2. Based on the findings, there was a significant difference between the treatment method and the prescribed drug type (P = 0.001). A significant association was found between the therapeutic approach and the treatment regimen (P = 0.001).

**Table 2:** Prescribed antibiotic regimen based on the patients’ treatment.

**The prevalence of prescription antibiotics**	**Hospitalization therapy**	**Outpatient therapy**
Ceftriaxone or cefotaxime	20 (85.8%)	112 (69.6%)
Ceftriaxone + clindamycin	5 (41.7%)	3 (2.6%)
Ceftriaxone + amikacin	5 (41.7%)	0 (0%)
Clindamycin	1 (2.9%)	1 (0.9%)
Vancomycin + amikacin	1 (2.9%)	0 (0%)
Other cases	2 (5.9%)	0 (0%)
**Continued treatment regimen**		
First line the antibiotic therapy	27 (97.4%)	116 (100%)
Change to vancomycin	3 (8.8%)	0 (0%)
**Change with antibiotics other than vancomycin**	4 (11.8%)	0 (0%)

Of the two cases with documented sepsis, one case was treated with ceftriaxone or cefotaxime, and other case was treated with ceftriaxone plus clindamycin; furthermore, none had received vancomycin therapy. In the follow-up period, none of the patients had an acute postoperative infection or OPSI.

## Discussion

What should be considered in the evaluation and management of patients with beta-thalassemia and the candidates for splenectomy, is to cover vaccination against infections such as pneumococcus, as well as the implementation of antibiotic prophylaxis.

In the past, the main directions of this prophylaxis were administration of penicillin, cephalosporins, or macrolides. In cases where there was hypersensitivity to these antibiotics, administration of vancomycin was considered as the second choice. According to recently published results, adding vancomycin to ceftriaxone or cefotaxime increases the antibiotic coverage in patients undergoing splenectomy and candidates for antibiotic prophylaxis that routinely received penicillin. This is particularly evident in pneumococcal strains resistant to various antibiotics [16]. Nevertheless, what is seen in the existing guidelines is that the drug was changed with vancomycin only due to penicillin and cephalosporin hypersensitivity. However, our findings indicated that only 2.7% of the patients were treated with vancomycin, and the antibiotic coverage in these patients was basically based after the administration of ceftriaxone or cefotaxime (in 88% of cases).

On the other hand, none of the two patients with definitive sepsis required treatment with vancomycin. The vast majority of patients treated with drugs other than vancomycin were mostly ceftriaxone or cefotaxime, which led to successful prophylaxis. Therefore, it can be concluded that the coverage of all types of sepsis-induced infections in patients with splenectomy, particularly streptococcus and pneumococcus, is sufficient through the combination of the above-mentioned drugs, and there is virtually no need to change the regimen of the drug with vancomycin.

Given that most of the patients were treated as an outpatient and did not need to be hospitalized, there is no need to add vancomycin to the prophylactic drug regimen, except for severe cases.

A 100% coverage of vaccination with antibiotic prophylaxis was observed in all thalassemia patients undergoing splenectomy, which was lower in various studies. In a study by Jones et al., 92% of the patients had received appropriate antibiotic therapy, but unfortunately, only 33% of the patients continued this antibiotic prophylaxis, which was, of course, limited to patients with low risk [17]. Antibiotic prophylaxis rates for the emergency cohort have been reported to be 88.9% as compared to 93.4% for the elective cohort [18]. Ramachandra et al. showed that only 72% of the patients were vaccinated, and 63% had been discharged with antibiotic prophylaxis, which was significantly lower than our study [19].

Another study showed that 86% of the patients were vaccinated against pneumococcus, and only 8.74% of patients had antibiotic prophylaxis. Only 4.37% of patients received both antibiotic vaccination and prophylaxis protocols simultaneously [20], which was very different from the protocol performed in our center. Waghorn and colleagues reported a high prevalence of OPSI with total mortality of 50% for these patients, due to the small coverage of antibiotic prophylaxis in this group of patients [21].

What should be considered as a general guide for a specialist is the following: first, the complete coverage with pneumococcal vaccination and antibiotic prophylaxis for patients with thalassemia major undergoing splenectomy; second, the addition of vancomycin to broad-spectrum antibiotics, especially cephalosporins, to the prophylactic antibiotic regime in these patients, leads to a minimization of the risk of OPSI. In our study, 100% coverage with vaccination and antibiotic prophylaxis was found in patients with thalassemia major undergoing splenectomy. The primary prophylactic antibiotic regimen included ceftriaxone or cefotaxime, and the coverage of vancomycin in combination with amikacin was observed in 0.7% cases, while a change of the drug regimen with vancomycin was seen in only 2.7% cases. Considering that the majority of patients received outpatient treatment, and there was no need for hospitalization except for severe cases of the disease; also, the addition of vancomycin to the prophylactic drug regimen was not necessary.

## Conclusion

Our results show that treatment perceptions in case of fever in splenectomized children need to be changed, and most of the cases do not require hospitalization and initiation of broad-spectrum antibiotics such as vancomycin for initially refractory cases, and can only be treated with daily intravenous ceftriaxone.

## Conflict of Interest

The authors declare that there is no conflict of interest.
